# MyD88 Shapes Vaccine Immunity by Extrinsically Regulating Survival of CD4^+^ T Cells during the Contraction Phase

**DOI:** 10.1371/journal.ppat.1005787

**Published:** 2016-08-19

**Authors:** Huafeng Wang, Mengyi Li, Chiung Yu Hung, Meenal Sinha, Linda M. Lee, Darin L. Wiesner, Vanessa LeBert, Tassanee Lerksuthirat, Kevin Galles, Marulasiddappa Suresh, Anthony L. DeFranco, Clifford A. Lowell, Bruce S. Klein, Marcel Wüthrich

**Affiliations:** 1 Departments of Pediatrics, University of Wisconsin School of Medicine and Public Health, University of Wisconsin-Madison, Madison, Wisconsin, United States of America; 2 Department of Biology and South Texas Center for Emerging Infectious Diseases, University of Texas at San Antonio, San Antonio, Texas, United States of America; 3 Department of Laboratory Medicine, University of California at San Francisco, San Francisco, California, United States of America; 4 Department of Microbiology and Immunology, University of California at San Francisco, San Francisco, California, United States of America; 5 Department of Pathobiological Sciences, University of Wisconsin School of Medicine and Public Health, University of Wisconsin-Madison, Madison, Wisconsin, United States of America; 6 Internal Medicine, University of Wisconsin School of Medicine and Public Health, University of Wisconsin-Madison, Madison, Wisconsin, United States of America; 7 Medical Microbiology and Immunology, University of Wisconsin School of Medicine and Public Health, University of Wisconsin-Madison, Madison, Wisconsin, United States of America; Rutgers Biomedical and Health Sciences, UNITED STATES

## Abstract

Soaring rates of systemic fungal infections worldwide underscore the need for vaccine prevention. An understanding of the elements that promote vaccine immunity is essential. We previously reported that Th17 cells are required for vaccine immunity to the systemic dimorphic fungi of North America, and that Card9 and MyD88 signaling are required for the development of protective Th17 cells. Herein, we investigated where, when and how MyD88 regulates T cell development. We uncovered a novel mechanism in which MyD88 extrinsically regulates the survival of activated T cells during the contraction phase and in the absence of inflammation, but is dispensable for the expansion and differentiation of the cells. The poor survival of activated T cells in *Myd88* ^-/-^ mice is linked to increased caspase3-mediated apoptosis, but not to Fas- or Bim-dependent apoptotic pathways, nor to reduced expression of the anti-apoptotic molecules Bcl-2 or Bcl-xL. Moreover, TLR3, 7, and/or 9, but not TLR2 or 4, also were required extrinsically for MyD88-dependent Th17 cell responses and vaccine immunity. Similar MyD88 requirements governed the survival of virus primed T cells. Our data identify unappreciated new requirements for eliciting adaptive immunity and have implications for designing vaccines.

## Author Summary

Despite several million new systemic fungal infections annually worldwide, there are no commercial vaccines available. The development of effective vaccines requires a fundamental understanding of how protective immune responses are induced. Using experimental vaccine strains, we previously demonstrated that populations of T helper cells producing interleukin 17 (Th17 cells) and interferon gamma (Th1 cells) mediate vaccine resistance to systemic dimorphic fungi of North America. Here, we report how the immune system recognizes the fungal vaccines and induces the development of protective T cells. We delineate the role of pathogen recognition receptors (PRRs) and their common signaling pathway in host immune cells that recognize the fungal vaccine. While the signaling pathway studied is essential for the development of vaccine-induced T cells, the mechanism of action is novel and included T cell death after activation. The findings could be extended to virus-specific T cells suggesting that the mechanism is conserved among the microbial kingdom. Our work sheds new light on how protective T cells are induced and can be harnessed by vaccine strategies tailored against fungal and other microbial infections.

## Introduction

The soaring rates of systemic fungal infections worldwide have spurred interest in developing vaccines [1,2,3,4,5,6,7]. We and others have engineered vaccines that protect against experimental infection with the primary pathogenic fungi *Coccidioides posadasii* [8], *Histoplasma capsulatum* [9] and *Blastomyces dermatitidis* [10], which cause the major systemic mycoses of North America and account for an estimated one million new infections annually [11]. CD4^+^ T cells are the primary effector cells that control fungal infections in healthy hosts [12,13] and Th17 cells are requisite for vaccination against the endemic mycoses of North America [14]. Vaccine induced Th17 cells confer resistance independent of Th1 cells by recruiting and activating neutrophils and macrophages to the alveolar space to reduce the burden of infection.

The development of effective vaccines requires a fundamental understanding of how protective immune responses are induced. We previously reported that the differentiation of Th17 cells and acquisition of vaccine immunity requires innate recognition and signaling through Card9 and MyD88 [14,15]. The innate immune system senses invading microbes through germline-encoded pattern-recognition receptors (PRRs) that bind conserved and invariant structures, termed pathogen-associated molecular patterns (PAMPs) [16]. Fungal PAMPs such as the cell-wall components chitin, α- and β-glucans, and mannans are unique to fungi and distinguish them from the host [17]. The PRRs that are best described for the recognition of fungi include the C-type lectins and Toll-like receptors (TLRs). Vaccination with *B*. *dermatitidis* requires Dectin-2 recognition and signaling for the development of Th17 cells [15], whereas the related dimorphic fungi *H*. *capsulatum* and *C*. *posadasii* require Dectin-1 and Dectin-2 for the induction of protective Th17 cell responses. Most TLRs (except for TLR3) and IL-1R family members trigger pathways via the adaptor protein myeloid differentiation primary-response gene 88 (MyD88) to activate NF-κB and mitogen-activated protein kinases (MAPK) [18,19].

While TLRs and MyD88 have been implicated in the development of Th1 and Th2 cells [20,21,22,23], their role in inducing Th17 cells is unexpected and poorly understood. The regulation of Th1 and Th2 cells by MyD88 is linked to TLR-dependent cytokine production by antigen presenting cells (APCs) that influence T cell differentiation [20,21,22,23].

Both T cell-extrinsic and -intrinsic MyD88 signaling promotes adaptive immune responses. T cell-extrinsic signaling activates dendritic cells (DCs) and macrophages to produce pro-inflammatory cytokines and promote antigen presentation to initiate adaptive immunity during viral, bacterial and parasitic infections [24]. Impaired MyD88 signaling increases susceptibility to fungal infections such as candidiasis, cryptococcosis, aspergillosis, paracoccidioidosis, pneumocystis and coccidioidomycosis [25,26,27]. However, the mechanisms by which MyD88 mediates adaptive immunity are not well understood. In addition to the extrinsic role of MyD88 signaling in immunity to fungal infections, T cell- intrinsic expression of MyD88 is required for resistance to infections with *Toxoplasma gondii*, *LCMV* and *B*. *dermatitidis*. In experimental *T*. *gondii* infection, T cell-intrinsic MyD88 is required for Th1 mediated resistance independent of IL-1R and IL-18R signaling, implying a role for TLRs [28]. During LCMV infection, IFN-γ-producing CD8^+^ T cells require intrinsic MyD88 signals for differentiation and survival [29]. Finally, CD8 T cell-intrinsic MyD88 signals are required for Tc17 cell responses and immunity to *B*. *dermatitidis* infection [30].

In the current study, we uncovered a novel mechanism by which MyD88 enables the development of vaccine-induced anti-fungal Th17 cells and resistance to infection. Instead of regulating the production of priming cytokines by APCs that shape T cell differentiation [20,21,22,23], MyD88 extrinsically regulates the survival of CD4^+^ T cells during the contraction phase under non-inflammatory conditions. T cell-intrinsic MyD88 signals were largely dispensable for the development of anti-fungal CD4^+^ T cells. Moreover, TLR3, 7, and 9 served as the extrinsic upstream sensors and signaling receptors that initiate T cell survival signals under non-inflammatory conditions. Similar MyD88 requirements extrinsically governed the survival of virus-primed T cells, implying a general mechanism across microbial kingdoms.

## Results

### Vaccine immunity against systemic dimorphic fungi requires the adaptor MyD88

**Fig 1:**
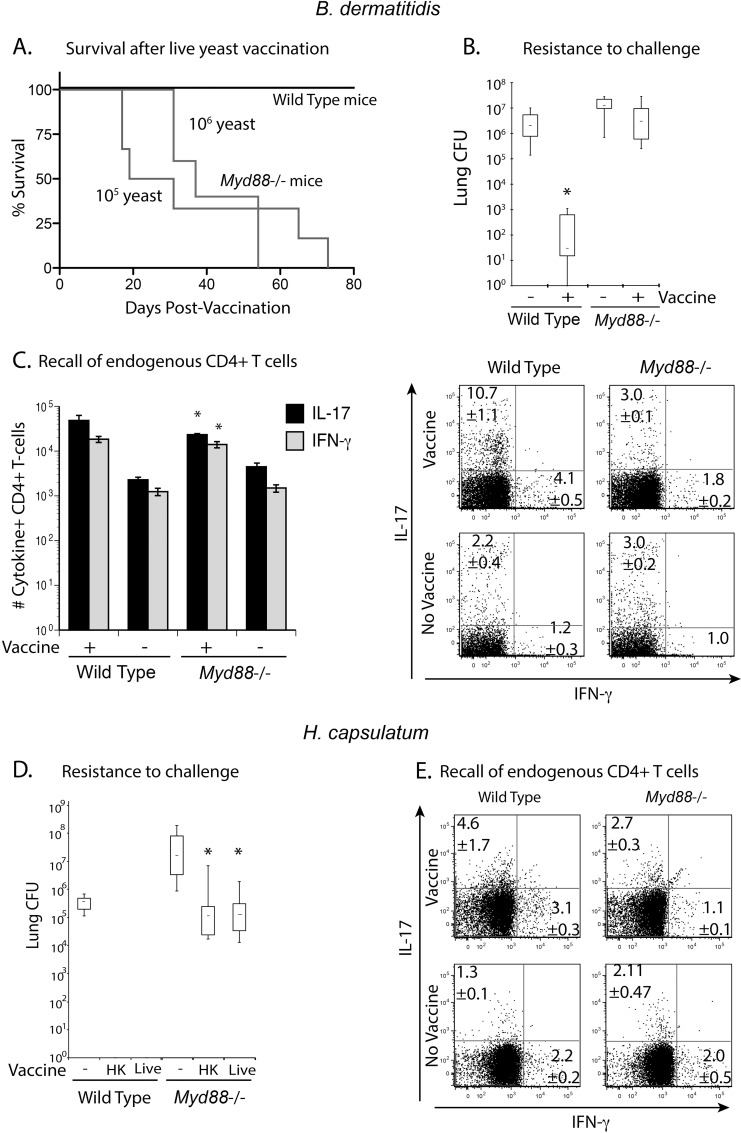
*Myd88* ^-/-^ mice fail to mount Th17 and Th1 cells and resistance after vaccination. Wild type C57BL6 and *Myd88* ^-/-^ mice were s.c. vaccinated with 10^5^ or 10^6^ live *B*.*dermatitidis* yeast and followed for survival **(A)**. To circumvent dissemination, mice were vaccinated s.c. with 10^6^ heat-killed yeast and challenged i.t. with 2 x 10^3^ 26199 yeast. Two weeks post-infection, lung CFU were enumerated **(B).** Data are the mean ± SEM (n = 8–10 mice/group). Data are representative of three independent experiments. The number of Th17 and Th1 cells recalled to the lung were assessed at day 4 post-infection. Data are the mean ± SEM (n = 4–6 mice/group) **(C).** The numbers indicate the mean ± SEM of cytokine producing CD4^+^ T cells per mouse. * *P* <0.05 vs. vaccinated wild-type controls. Dot plots show concatenated samples of 4–6 mice/ group. Data are representative of three independent experiments. **(D)** Wild-type and *Myd88* ^-/-^ mice were vaccinated s.c. with 10^7^ live *H*. *capsulatum* yeast and challenged with 5 x 10^5^ Hc G217B yeast. Two weeks post-infection, lung CFU were enumerated. * *P* <0.05 vs. vaccinated wild-type controls. **(E)** The frequencies of Th17 and Th1 cells were enumerated at day 4 post-infection. Data are the mean ± SEM (n = 4–6 mice/group).

We previously reported that vaccine-induced Th17 and Th1 cells were necessary and sufficient to protect mice against the three major systemic mycoses in North America [12,14]. *Myd88* ^-/-^ mice are highly susceptible to primary infections by *B*. *dermatitidis*, *P*. *brasiliensis*, *A*. *fumigatus*, *C*. *neoformans*, and *C*. *albicans* [14,31,32,33]. Here, we investigated whether *Myd88* ^-/-^ mice can acquire vaccine immunity against infection with the systemic dimorphic fungi *B*. *dermatitidis* and *H*. *capsulatum*. *Myd88* ^-/-^ mice were unable to control the live, attenuated #55 vaccine strain of *B*. *dermatitidis* and succumbed to dissemination and infiltration of the lungs by the yeast (Fig 1A). To circumvent susceptibility of *Myd88* ^-/-^ mice to the vaccine strain, we immunized them with heat-killed yeast and tested their ability to resist a lethal pulmonary infection with wild-type yeast. Vaccinated *Myd88* ^-/-^ mice failed to acquire resistance and had similar numbers of lung CFU when compared to unvaccinated littermates, which was 4–5 logs higher than in vaccinated wild-type control mice (Fig 1B). Vaccinated *Myd88* ^-/-^ mice had significantly fewer numbers and frequencies of endogenous Th1 and Th17 cells in their lungs on recall than did vaccinated wild-type controls (Fig 1C), which correlated with reduced resistance. To investigate whether these findings apply to other systemic dimorphic fungi, we vaccinated mice with *H*. *capsulatum*. Although *Myd88* ^-/-^ mice were able to control vaccination with live *H*. *capsulatum* yeast, they were significantly less resistant than vaccinated wild-type controls to lethal pulmonary challenge (Fig 1D). Likewise, vaccinated *Myd88* ^-/-^ mice recruited lower frequencies of endogenous Th17 and Th1 cells to the lung on recall (Fig 1E), which again correlated with reduced resistance. Thus, vaccinated *Myd88* ^-/-^ mice fail to recruit endogenous Th17 and Th1 cells to the lung, which are required to confer vaccine-induced resistance to systemic dimorphic fungi.

### T cell-intrinsic MyD88 is dispensable for the development of Th17 and Th1 cells

**Fig 2:**
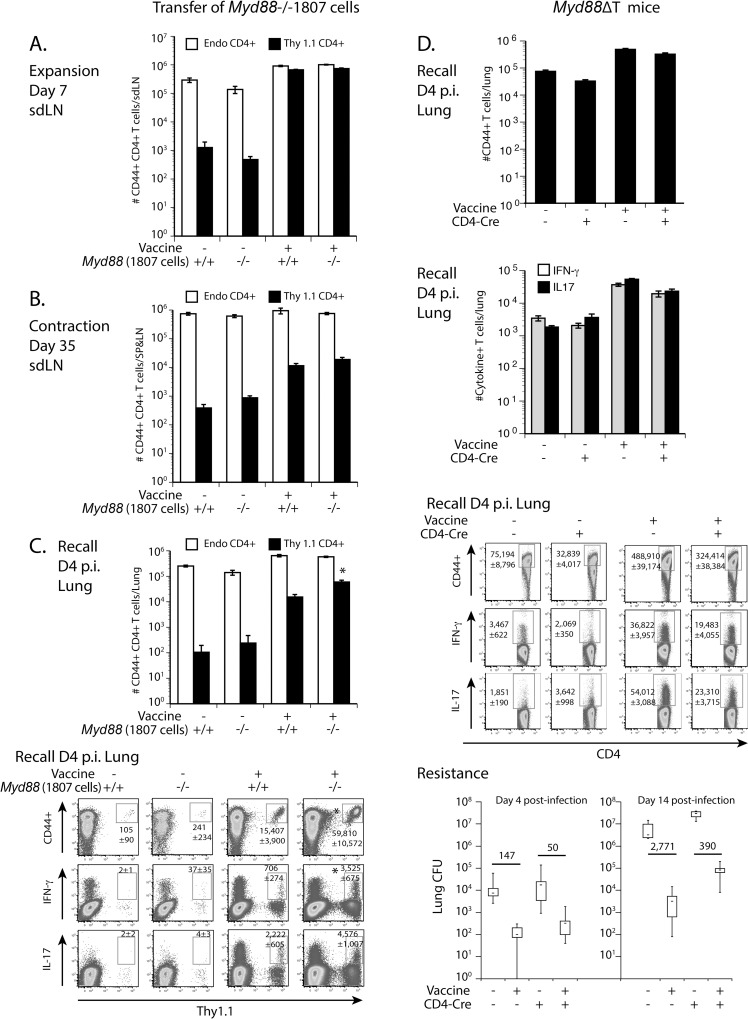
Intrinsic MyD88 is dispensable for the development of Th17 and Th1 cells. Naïve *Myd88* ^-/-^ and *Myd88* ^+/+^ 1807 T cells (indicated by -/- and +/+) were adoptively transferred into wild type C57BL6 recipient mice prior to s.c. vaccination (Panels A-C). Expansion **(A)**, contraction **(B)** and migration **(C)** of transferred 1807 cells and endogenous CD4^+^ T cells was determined by FACS in the skin draining lymph nodes (sdLN) on days 7 and 35 post-vaccination and in the lung at day 4 post-infection. Data are representative of five independent experiments. * *P* < 0.05 vs. wild type 1807 T cells. **(D)** *Myd88*∆T mice that are CD4-Cre^+^ *Myd88*fl/fl lack MyD88 in αβT cells and CD4-Cre^-^ *Myd88*fl/fl controls were vaccinated with 10^6^ heat-killed vaccine yeast and challenged with wild-type yeast. The number of activated (CD44^+^) and cytokine producing CD4^+^ T cells were enumerated at day 4 post-infection. Lung CFU were determined at day 4 and two weeks post-infection. Data from day 4 post-infection are an average of three independent experiments and day 14 post-infection data are representative of two independent experiments. The numbers in the graph indicate the n-fold change in lung CFU vs. unvaccinated control mice.

T cell-intrinsic MyD88 is necessary for resistance to *LCMV* and *T*. *gondii* infections [28,29,34] and survival of CD8^+^ T cells [28,29,35]. To investigate whether MyD88 affects the development of vaccine induced CD4^+^ T cells in a T cell-intrinsic manner, we used two approaches. First, we generated *B*. *dermatitidis*-specific TCR Tg (1807) mice that lack MyD88, as described in the methods. We adoptively transferred naïve Tg T-cells from these mice into wild-type recipients before subcutaneous vaccination. Thus, recipient host APCs are wild-type and transferred fungus-specific CD4^+^ T cells lack MyD88. As a positive control, we also transferred naïve wild-type 1807 into wild-type recipients before vaccination. At serial time intervals post-vaccination, we analyzed the behavior of the two transferred 1807 T cell populations. The number of activated (CD44^+^), *Myd88* ^-/-^ vs. *Myd88* ^+/+^ 1807 cells was not reduced at day 7 post-vaccination (burst of T cell expansion) (Fig 2A), at day 35 (contraction of T cells) (Fig 2B) and at day 4 post-infection (recall to the lung) (Fig 2C). Likewise, cytokine production by *Myd88* ^-/-^ vs. *Myd88* ^+/+^ 1807 cells in the lung after recall was not reduced (Fig 2C). Thus, MyD88 does not intrinsically regulate the development of CD4^+^ T cells in our vaccine model.

We sought to validate the results observed with TCR Tg cells by also investigating polyclonal, endogenous CD4^+^ T cells. Here, we vaccinated *Myd88*∆T mice in which MyD88 expression is absent only in αβT cells [35], and enumerated the number of cytokine producing lung CD4^+^ T cells upon recall. The numbers of activated (CD44^+^) and cytokine-producing T cells were similarly increased in vaccinated *Myd88*∆T and wild-type control mice, as compared to unvaccinated mice (Fig 2D). Vaccinated *Myd88*∆T mice acquired resistance similar to wild-type controls at day 4 post-infection, although resistance was modestly reduced in the *Myd88*∆T mice at two weeks post-infection (Fig 2D). Thus, T cell-intrinsic MyD88 may have a small (if any) impact on vaccine resistance mediated by CD4^+^ T cells in our model, but it does not explain the profound impairment in resistance mediated by the cells in vaccinated *MyD88* ^-/-^ mice.

### Extrinsic MyD88 is required for the survival of activated T cells

**Fig 3:**
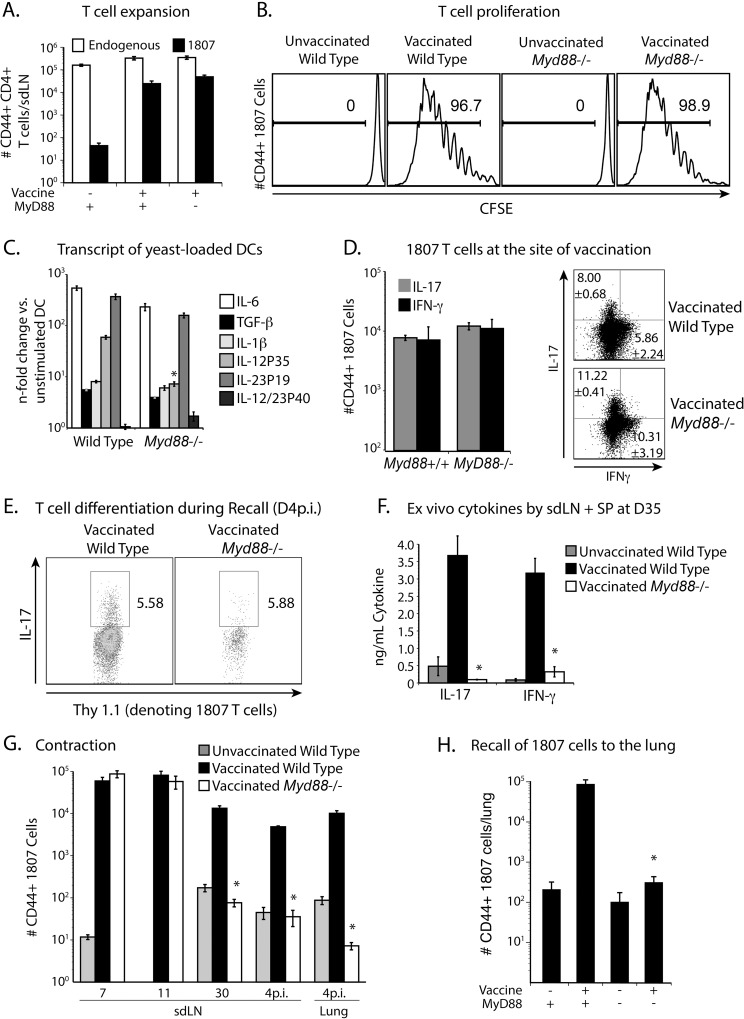
Extrinsic MyD88 regulates T cell contraction. **(A)** 10^6^ CD4^+^ purified, CFSE-labeled, naïve 1807 Tg cells were transferred into wild type and *Myd88* ^-/-^ mice that were then vaccinated s.c. with 10^6^ heat-killed vaccine yeast or not. At day 7 post-vaccination, the skin draining lymph nodes (sdLN) were harvested and the numbers of activated (CD44^+^) 1807 T cells and endogenous CD4^+^ T-cells enumerated by FACS. **(B)** T cell proliferation of 1807 T cells is indicated by CFSE loss. **(C)**. BMDC from wild type and *Myd88* ^-/-^ mice were co-cultured with vaccine yeast for 24 h and transcript analyzed by RT-PCR. * *P* < 0.05 vs. increase in transcript in wild type DC. **(D)** At day 11 post-vaccination, the frequency of cytokine producing 1807 T cells was determined at the site of vaccination. Dot plots show the sum of concatenated events from 6 mice/group and the numbers indicate the mean ± SEM of cytokine producing 1807 T cells. **(E)** Upon recall at day 4 post-infection, the frequency of IL-17 producing 1807 T cells was quantified in the lung. Data are representative of two independent experiments. **(F)** At day 35 post-vaccination, endogenous CD4^+^ T cells were purified from the skin draining lymph nodes (sdLN) and spleen of vaccinated wild type and *Myd88* ^-/-^ mice and stimulated with CW/M antigen for 3 days. Cytokines were measured in the cell-culture supernatants by ELISA. *P < 0.05 vs. cytokine production by CD4^+^ T cells from vaccinated wild type controls. **(G, H)** 10^6^ purified naïve CD4^+^ 1807 T cells were transferred into wild type and *Myd88* ^-/-^ mice that were vaccinated s.c. with 10^6^ heat-killed vaccine yeast or not. At serial time points post-vaccination, the sdLN and lung were harvested and the number of CD44^+^ 1807 T cells enumerated by FACS. * *P* < 0.05 vs. number of activated CD4^+^ T cells from vaccinated wild type controls. Data are representative of three independent experiments.

To investigate whether extrinsic MyD88 is required for the development of Th17 and Th1 cells, we adoptively transferred purified, naïve CD4^+^ T cells from wild-type 1807 mice into *Myd88* ^-/-^ and *Myd88* ^+/+^ recipients before subcutaneous vaccination. At serial intervals post-vaccination, we compared development of wild-type 1807 cells in the two recipient hosts. T cell expansion, activation and proliferation of 1807 cells were similar in the recipient hosts, as indicated by the number of CD44^+^ 1807 T cells and loss of CFSE (Fig 3A and 3B).

To assess T cell differentiation, we used several approaches. Since T cell differentiation is orchestrated by the cytokine milieu produced by APCs, we first determined the n-fold change in cytokine transcripts after co-culture with vaccine yeast. The n-fold increase in the Th17 (IL-6, TGF-β, IL-1β and IL-23p19) and Th1 (IL-12p35) priming cytokines was comparable with bone marrow DCs from *Myd88*-deficient and -sufficient mice (Fig 3C). Second, we determined the frequencies of Th17 and Th1 1807 T cells at the site of vaccine injection (subcutaneous tissue) at day 11 post-vaccination and upon recall of cells to the lung at day 4 post-infection. The frequencies of cytokine-producing 1807 T cells (and numbers at vaccine injection site) were comparable in both strains of vaccinated recipients (Fig 3D and 3E). Third, we stimulated primed endogenous CD4^+^ T cells *ex vivo* with CW/M antigen and measured cytokines in the cell-culture supernatant. The amount of IL-17 and IFN-γ produced by T cells from vaccinated *Myd88* ^-/-^ mice was significantly reduced compared to the cells from wild type mice (Fig 3F).

Since reduced cytokine production by endogenous T cells could be due to either a lack of intrinsic MyD88 or reduced numbers of antigen-experienced T cells after contraction, we enumerated activated (CD44^+^) T cells in *Myd88* ^-/-^ and wild type mice serially after vaccination. For this, we transferred 1807 cells into both recipients. We found that by 30 days post-vaccination the number of activated 1807 T cells was significantly reduced in the skin draining lymph nodes (sdLN) and in the lung upon recall (Fig 3G and 3H), indicating enhanced contraction when MyD88 is lacking extrinsically (in APCs). These data suggest that MyD88 extrinsically regulates the survival of activated CD4^+^ T cells, but does not affect their activation, expansion, proliferation or differentiation.

### MyD88 extrinsically promotes T cell survival during the contraction phase and not the programming phase

**Fig 4:**
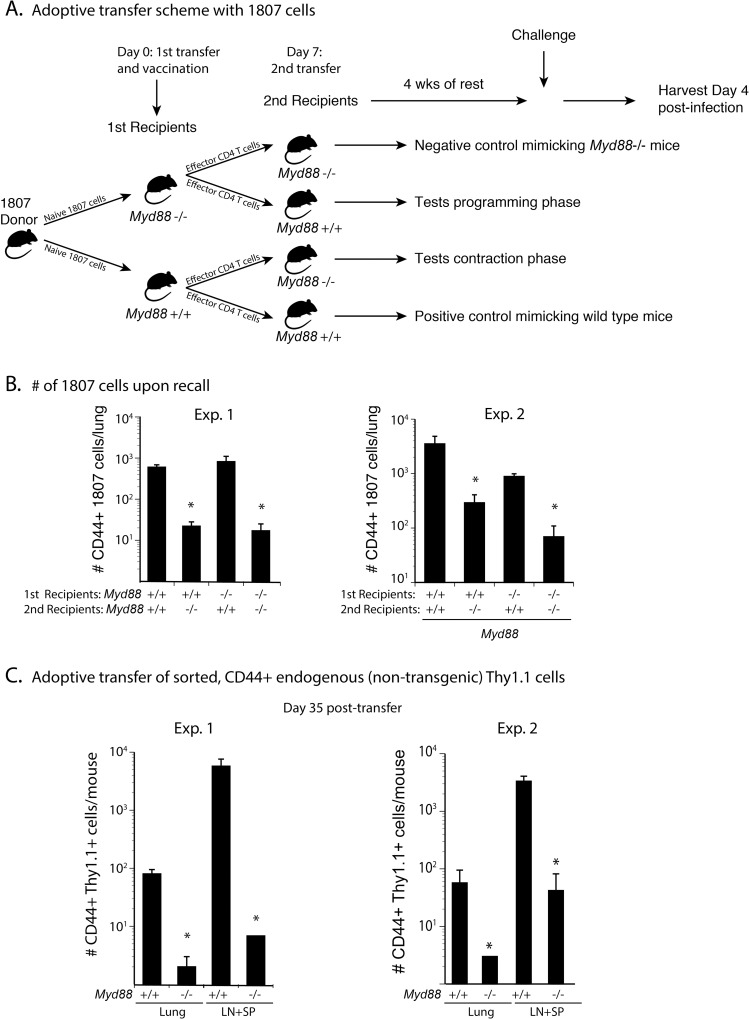
Extrinsic MyD88 regulates T cells during the contraction phase, but not the programming phase. **(A)**. Schematic: One million purified CD4^+^ T cells from naïve 1807 mice were transferred into *Myd88* ^-/-^ and wild type mice prior to vaccination. Seven days post-vaccination, CD4^+^ T cells were magnetic bead-purified from the sdLN and spleen of the vaccinated mice and adoptively transferred into new sets of naïve *Myd88* ^-/-^ and wild type mice as shown in the schematic and activated effector T cells were rested for 4wks. **(B)** Recipient mice were challenged and 1807 T cells enumerated in the lung at day 4 post-infection. Data are representative of two independent experiments. * *P* < 0.05 vs. groups with no asterisk. **(C)** Congenic Thy1.1^+^ wild type mice were vaccinated and at day 7 post-vaccination the CD4^+^ T were sorted for CD44^+^ T cells, transferred into naïve *Myd88* ^-/-^ and wild type mice, and the mice rested for 4 wks as above. At day 4 post-challenge, the numbers of congenic Thy 1.1^+^ T cells were determined by FACS. Data are the mean ± SEM (n = 4–6 mice/group). Data are representative of two independent experiments. * *P* < 0.05 vs. corresponding wild type controls.

Since MyD88 extrinsically regulates T cell survival and not activation or differentiation (Fig 3G and 3H) we investigated whether it programmed activated T cells during the expansion phase (to make them fit to survive contraction) or exclusively during the contraction phase. To test these two possibilities, we adoptively transferred naïve wild type 1807 cells into *Myd88*-deficient and–sufficient recipient mice prior to vaccination (Fig 4A). At day 7 post-vaccination, we harvested and sorted CD44^+^ CD4^+^ T cells from the sdLN and spleen of these initial recipients, adoptively transferred them into new sets of naïve *Myd88* ^-/-^ and wild type mice and let the activated T cells rest for 4 weeks. To accumulate the transferred T cells for analysis, we challenged the mice and enumerated 1807 T cells recalled to the lung at day 4 post-infection. The number of CD44^+^ 1807 T cells was high when MyD88 was present during the contraction phase (Fig 4B). These results also indicate that MyD88 promotes T cell survival under homeostatic, resting conditions in the absence of vaccine-induced inflammation. When MyD88 was absent during the contraction phase, the number of activated 1807 cells was as low as when MyD88 was missing throughout the experiment. The absence of MyD88 during the expansion/programming phase did not affect T cell survival. Thus, MyD88 is dispensable during the first seven days post-vaccination and required during the contraction.

We next studied endogenous cells to validate our findings with TCR Tg 1807 T cells. We harvested lymph nodes and spleens from vaccinated wild type Thy1.1^+^ mice at the peak of expansion and transferred sorted CD44^+^ CD4^+^ T cells into naïve wild type and *Myd88* ^-/-^ mice. After four weeks of rest, the transferred T cells were recalled into the lung (with challenge) and enumerated in the lymph nodes and spleen. The number of transferred CD44^+^ CD4^+^ T cells was reduced in the lung, lymph nodes and spleen of *Myd88* ^-/-^ recipient vs. wild type recipient mice (Fig 4C). Thus, MyD88 extrinsically regulates the survival of activated CD4^+^ T cells during the contraction phase.

### The protective effects of MyD88 are due to reduced apoptosis, but not the expression of survival factors

**Fig 5:**
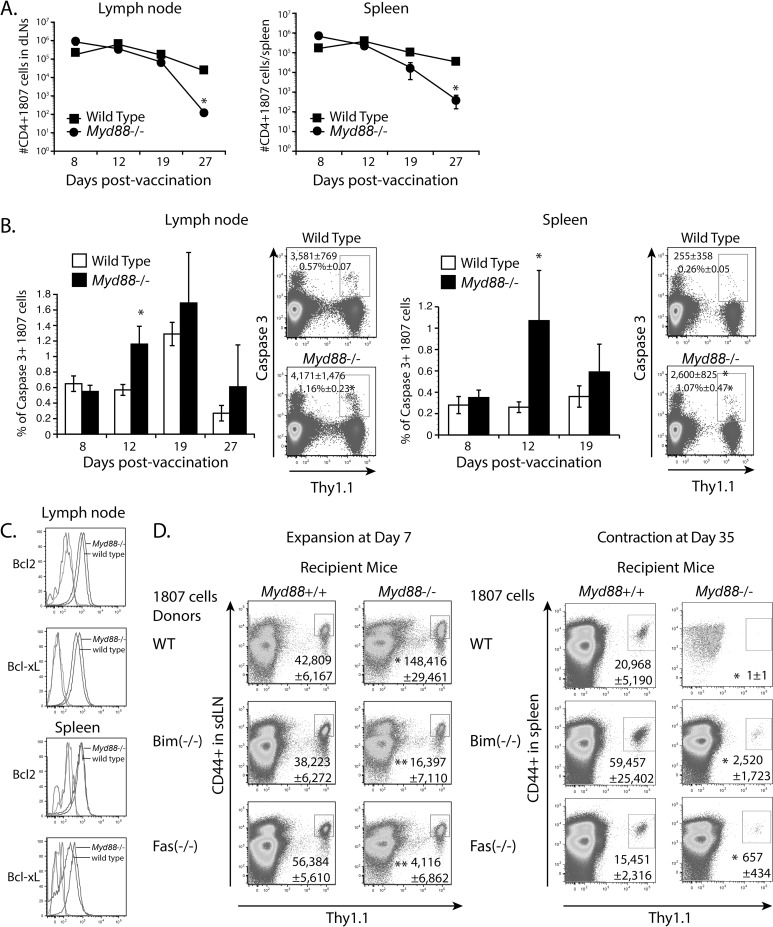
The role of extrinsic MyD88 for effector CD4^+^ T cell survival. **(A-C)** Mice received 10^6^ purified CD4^+^ T cells from naïve 1807 mice and were vaccinated with heat-killed yeast. **(A)** 1807 T cell contraction. At serial time intervals post-vaccination, the numbers of activated 1807 T cells were enumerated from the sdLN and spleen by FACS. * *P* < 0.05 vs. wild type control recipient mice. **(B)** Caspase 3 expression. Numbers in the dot plot indicate the numbers (top line) and frequencies (2^nd^ line from top) of intracellular caspase 3 positive 1807 T cells within the CD4^+^ T cell gate at day 12 post-vaccination. * *P* < 0.05 vs. wild type control recipient mice. **(C)** The mean fluorescence intensity of Bcl2 and Bcl-xL expression in 1807 T cells transferred into wild type and *Myd88* ^-/-^ recipients. Isotype controls are shown on the left of the graphs (unlabeled). **(D)** Adoptive transfer of naïve Bim^-/-^ 1807 T cells and Fas^-/-^ 1807 T cells into *Myd88* ^-/-^ and wild type controls prior to vaccination. At days 7 and 35 post-vaccination, the numbers of adoptively transferred 1807 T cells were enumerated in the sdLN and spleen. Data are the mean ± SEM (n = 4–6 mice/group). Data are representative of two independent experiments. * *P* < 0.05 vs. corresponding wild type recipient mice. ** *P* < 0.05 vs. corresponding wild type 1807 cells.

The net number of T cells during the contraction phase is largely governed by apoptosis of effector cells. Thus, we asked whether adoptively transferred 1807 T cells undergo enhanced apoptosis in *Myd88* ^-/-^ mice. At day 12 and 19 post-vaccination, which is a few days before we noted increased T cell contraction in *Myd88* ^-/-^ mice (Fig 5A), we found increased expression of active caspase 3, but not caspase 8, in activated 1807 T cells (Fig 5B). Because Bcl2 and Bcl-xL promote survival of effector T cells [36], we assessed whether increased contraction of activated CD4^+^ T cells in *Myd88* ^-/-^ mice is linked with reduced expression of the anti-apoptotic molecules Bcl-2 and Bcl-xL. The expression of both anti-apoptotic markers in transferred 1807 T cells was comparable in vaccinated wild type and *Myd88* ^-/-^ recipients at time points before the T cells underwent enhanced contraction in the latter group (Fig 5C). Thus, reduced 1807 T cell numbers in the absence of extrinsic MyD88 signaling is linked to enhanced apoptosis, but not reduced expression of Bcl-2 and Bcl-xL.

### Does MyD88 regulate T cell contraction through the extrinsic (Fas) or intrinsic (Bim) apoptotic pathway?

To determine whether MyD88 regulates T cell contraction through the extrinsic vs. intrinsic death pathway, we generated 1807 mice and T cells that lack Fas or Bim, respectively, and adoptively transferred wild type 1807 cells or crossed (pathway-deficient) 1807 cells into *Myd88* ^-/-^ and *Myd88* ^+/+^ recipient mice (S1A Fig). If MyD88 regulates T cell contraction through Bim, we would expect that the absence of Bim would repair the MyD88 phenotype and the number of Bim-deficient 1807 cells would be similar in the *Myd88* ^-/-^ and *Myd88* ^+/+^ recipients after contraction. If the number of Bim-deficient 1807 cells were lower in *Myd88*-deficient mice, this would indicate that MyD88 regulates contraction through a different pathway. Conversely, the same reasoning would hold true for Fas-deficient 1807 cells.

At day 7 post-vaccination, the expansion of all three lines of adoptively transferred 1807 cells was comparable in the sdLN of wild type and *Myd88* ^-/-^ mice (Fig 5D). At day 35 post-vaccination, the contraction of all three lines of 1807 cells was still enhanced in *Myd88*-deficient vs. *MyD88*-sufficient recipients, indicating either that MyD88 does not mediate enhanced contraction through the Bim or Fas apoptotic pathways, or alternatively that MyD88 regulated contraction occurs through both pathways, so that when one pathway is eliminated, the other pathway compensates.

### MyD88 does not regulate expression of the pro-survival cytokines IL-7, IL-15 and type I interferons in naïve mice

The IL-2 cytokine family members IL-7 and IL-15 promote the survival of naïve, activated and memory T cells [37,38] and type I interferons IFN-α/β prevent the death of activated T cells by directly acting on them [39]. To investigate whether MyD88 mediates T cell contraction by regulating the expression of IL-2 family members and type I IFNs, we determined cytokine transcripts in naïve mice. We found no significant reduction in transcripts of IL-7, IL-15, IFN-α, IFN-β (not detected), IFN-α receptor 1 (IFNAR1), IFNAR2, and interferon regulatory factory 8 (IRF8) (S1B and S1C Fig), which impacts type I IFN expression and function, in skin draining lymph node cells and splenocytes of *Myd88* ^-/-^ vs wild type mice. The master regulator of type I IFN expression IRF7 [40,41] was consistently and significantly reduced in naïve *Myd88* ^-/-^ vs. wild type mice (S1C Fig). However, the IRF7 phenotype was not sufficient to explain the T cell survival defect (see below).

### MyD88 in CD11c^+^ cells is dispensable for enhanced T cell contraction

Since antigen presentation and T cell stimulation is mostly conducted by professional, myeloid APCs that express CD11c on their surface, we hypothesized that CD11c^+^ cells are required to mediate MyD88-dependent T cell contraction. To test our hypothesis, we primed naïve 1807 T cells in vaccinated wild type recipient mice and adoptively transferred them into naïve CD11cCre^+^-*Myd88* ^fl/fl^ mice that lack MyD88 expression in CD11c^+^ cells and CD11cCre^—^ *Myd88* ^fl/fl^ controls. After four weeks of rest, we found no difference in 1807 T cell numbers from the two groups of recipient mice (S1F Fig) indicating that myeloid CD11c^+^ cells are dispensable for MyD88-dependent T cell contraction.

### TLR379 promote the survival of activated T cells

**Fig 6:**
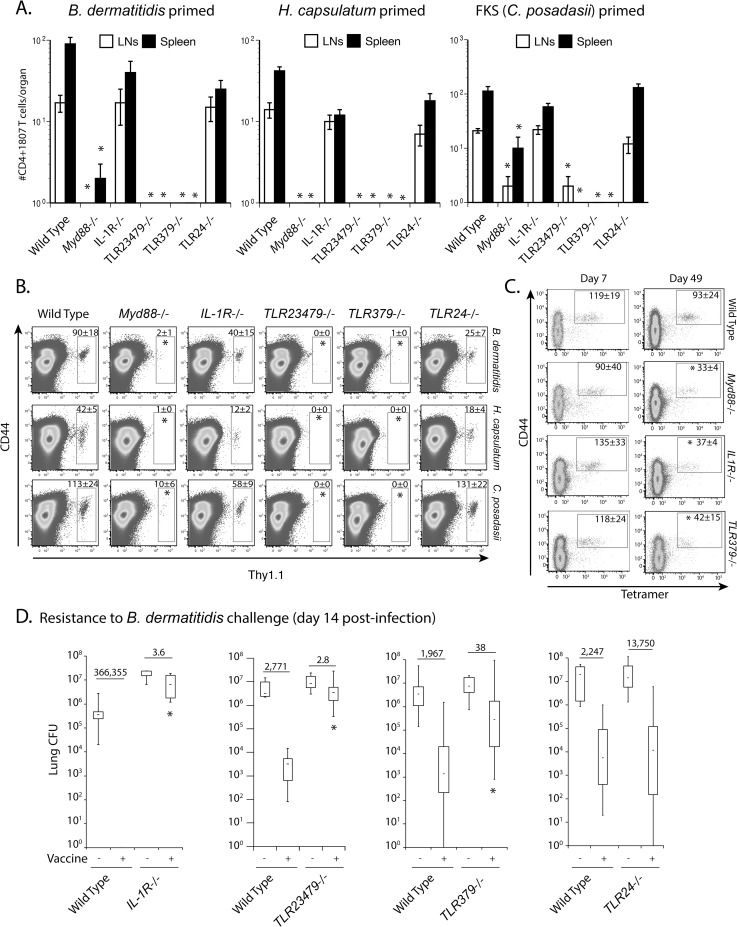
TLR3, 7 and 9 upstream of MyD88 extrinsically regulate T cell contraction. One million CD4^+^ T cells from naïve 1807 mice were transferred into wild type mice prior to vaccination. Seven days post-vaccination, effector CD4^+^ T cells were magnetic bead-purified from the sdLN and spleen of the vaccinated mice and adoptively transferred into naïve *Myd88* ^-/-^, IL-1R^-/-^, TLR23479^-/-^, TLR379^-/-^ and TLR24^-/-^ and wild type mice. After 4 weeks of rest, primed 1807 T cells harvested from the lymph nodes and spleen were enumerated by FACS. **(A)** Data are expressed as the mean ± SD of 8–12 mice/group. Data represent the average from two independent experiments. * *P* < 0.05 vs. wild type control mice. **(B)** shows the dot plots of concatenated events from 8–12 mice/group from panel A. * *P* < 0.05 vs. wild type control mice. **(C)** *Myd88* ^-/-^, IL-1R^-/-^, TLR23479^-/-^, TLR379^-/-^ and wild type mice were vaccinated with 10^6^ heat-killed vaccine yeast. At days 7 and 49 post-vaccination, the number of Cnx-specific CD4^+^ T cells in the sdLN and spleen was enumerated using tetramer enrichment and FACS detection. Tetramer positive cells are shown within the gate in each dot plot. The numbers represent the geometric mean ± SEM of tetramer-positive cells of 5 mice. Data are expressed from a single experiment representative of three independent experiments. * *P* < 0.05 vs. wild type control mice. **(D)** *Myd88* ^-/-^, IL-1R^-/-^, TLR23479^-/-^, TLR379^-/-^, TLR24^-/-^ and wild type mice were vaccinated as above, challenged and lung CFU enumerated at day 14 post-infection when unvaccinated mice were moribund. The numbers indicate the n-fold change vs. unvaccinated mice. Data are the mean ± SD of 10–20 mice/group from a single experiment representative of three independent experiments. * *P* < 0.05 vs. vaccinated wild type control mice.

MyD88 is an adaptor molecule that serves signaling of most TLRs and members of the IL-1R and IL-18R family. To investigate receptors upstream of MyD88 that extrinsically regulate contraction of T cells, we primed naïve 1807 cells in vaccinated wild type recipient mice with three systemic dimorphic fungi in parallel (*B*. *dermatitidis*, *H*. *capsulatum* and *C*. *posadasii*), purified CD4^+^ T cells from primary recipients and adoptively transferred them into mice that lack IL-1R; TLR2,3,4,7,9; TLR3,7,9; or TLR2,4 (as shown for MyD88 in Fig 4A). After four weeks of rest in all three fungal priming models, activated (CD44^+^) 1807 T cells were sharply diminished in TLR2,3,4,7,9^-/-^ and TLR3,7,9^-/-^ mice, similar to vaccinated *Myd88* ^-/-^ mice (Fig 6A and 6B). In IL-1R^-/-^ and TLR2,4^-/-^ mice the number of primed 1807 cells was reduced modestly in some cases, but it was not found to be statistically significant.

To investigate whether increased T cell contraction of effector T cells will yield reduced T cell numbers upon recall, we adoptively transferred naïve 1807 T cells into *Myd88* ^-/-^, IL-1R^-/-^, TLR2,3,4,7,9^-/-^, TLR3,7,9^-/-^, TLR2,4^-/-^ and wild type mice prior to vaccination. After recall, the number of primed 1807 T cells in the lung was reduced in vaccinated TLR2,3,4,7,9^-/-^ and TLR3,7,9^-/-^ vs. wild type mice (S2A and S2B Fig). In vaccinated IL-1R^-/-^, the recruitment to the lung and differentiation of activated 1807 T cells was also reduced compared to vaccinated wild type mice as indicated by the number of CD44^+^ 1807 cells (S2A and S2B Fig) and lower frequency of IL-17 producing 1807 cells (S2C Fig). As a consequence, vaccinated IL-1R^-/-^ mice failed to recruit Th17 cells to the lung upon recall (S2A and S2B Fig). Similar numbers of activated and cytokine producing 1807 T cells migrated to the lungs of vaccinated TLR2,4^-/-^ vs. wild type controls upon recall.

To validate the above results with Tg 1807 T cells, we analyzed endogenous, Ag-specific T cells and measured contraction using a recently generated tetramer that recognizes Calnexin (Cnx)-specific T cells in mice exposed to *B*. *dermatitidis* or related ascomycetes [42]. We vaccinated mice with heat-inactivated vaccine yeast and assessed the expansion and contraction of endogenous Cnx-specific CD4^+^ T cells. Tetramer^+^ CD44^+^ CD4^+^ T cells from vaccinated wild type and all knockout groups of mice expanded similarly, but contracted in an enhanced fashion in *Myd88* ^-/-^, TLR3,7,9^-/-^ and IL-1R^-/-^ mice (Fig 6C). Thus, the contraction of endogenous Cnx-specific T cells in these strains of mice mirrors the defect observed with activated 1807 T cells.

To determine whether the enhanced contraction of vaccine-induced T cells in these strains of mice impacts resistance, we assessed the burden of lung infection two weeks after challenge with *B*. *dermatitidis* and *C*. *posadasii*. Vaccinated TLR2, 3, 4, 7, 9^-/-^, TLR3, 7, 9^-/-^ and IL-1R^-/-^ mice had increased lung CFU compared to vaccinated wild type controls and TLR2,4^-/-^ mice (Fig 6D and S2D Fig). Thus, the number of vaccine-induced Ag-specific T cells that survive contraction forecasts resistance to fungal challenge on recall.

Since IRF7 expression is consistently reduced in *Myd88* ^-/-^ vs. wild type mice (S1C Fig) and TLR3, 7, 9^-/-^ and TLR2, 3, 4, 7, 9^-/-^ but not TLR2, 4^-/-^ mice showed enhanced T cell contraction (Fig 6A and 6B), we sought to investigate whether IRF7 transcripts are consistent with T cell survival in these strains of mice. If IRF7 regulates type I interferon expression and T cell survival downstream, then we would expect that IRF7 expression is similarly reduced in naïve TLR3, 7, 9^-/-^ and TLR2, 3, 4, 7, 9^-/-^ but not in TLR2, 4^-/-^ mice. In whole lymph node and spleen homogenates, IRF7 expression was reduced in naïve Myd88^-/-^, TLR3, 7, 9^-/-^, TLR2, 3, 4, 7, 9^-/-^, TLR2, 4^-/-^ vs. wild type mice (S1D Fig). The fact, that IRF7 expression is reduced in TLR2, 4^-/-^ vs. wild type mice is not compatible with the lack of enhanced T cell contraction in TLR2, 4^-/-^ mice. Since plasmacytoid DC (pDC) are the professional type I interferon producing DC subset, we sought to investigate whether IRF7 expression in pDC is compatible with the T cell survival phenotype in the knockout vs. wild type mice. Thus, we negatively enriched pDC from the spleen and analyzed IRF7 expression by real time RT-PCR. IRF7 expression in pDC was reduced in Myd88^-/-^ vs. wild type mice but not in TLR3, 7, 9^-/-^ and TLR2, 4^-/-^ mice (S1E Fig). The lack of reduced IRF7 expression in TLR3, 7, 9^-/-^ vs. wild type mice is not compatible with enhanced T cell contraction in these mice. In sum, we were able to exclude IRF7 and type I IFNs as the pro-survival signal for activated T cells.

### TLRs and IL-1R promote T cell survival via MyD88 signaling after priming with LCMV

**Fig 7:**
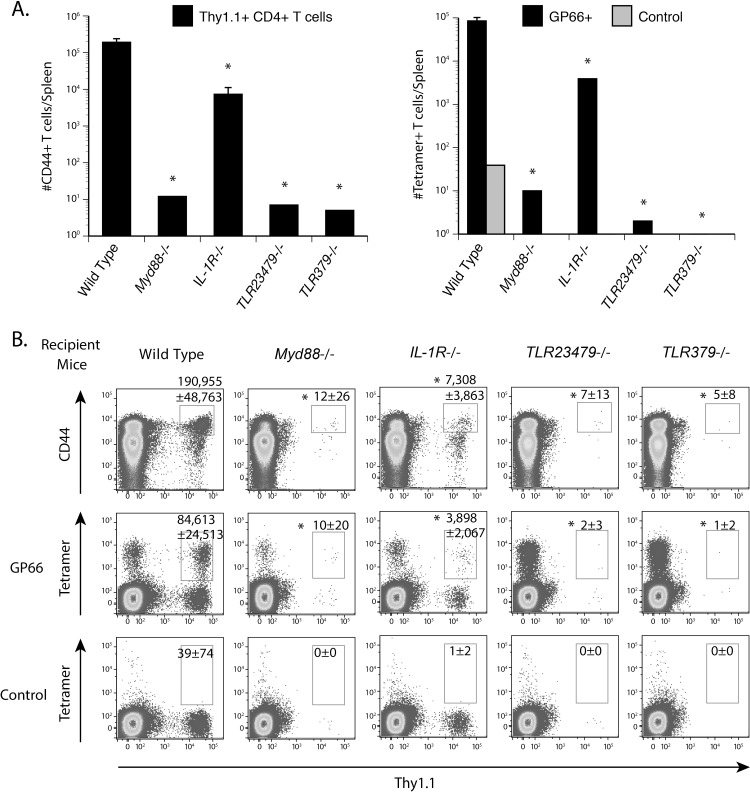
TLR379 upstream of MyD88 extrinsically regulate contraction of LCMV primed CD4^+^ T cells. Congenic Thy 1.1^+^ wild type mice were infected with 2 x 10^5^ pfu of LCMV Armstrong. At day 8 post-infection, CD4^+^ T cells were purified from the spleen and adoptively transferred into naïve *Myd88* ^-/-^, IL-1R^-/-^, TLR23479^-/-^, TLR379^-/-^, TLR24^-/-^ and wild type mice. After 4 weeks of rest, tetramer-positive, gp66-specific CD4^+^ T cells were enumerated in the spleen. **(A, B)** The numbers represent the geometric mean ± SEM of tetramer-positive cells from 5 mice. Data are expressed from a single experiment representative of two independent experiments. * *P* < 0.05 vs. vaccinated wild type control mice.

To investigate whether our findings are specific to vaccination with fungi or apply to other classes of microbes, we studied the contraction of LCMV primed CD4^+^ T cells after infection. We elicited LCMV primed T cells in congenic wild type mice, purified CD4^+^ T cells on day 8 post-infection at the peak of T cell expansion, and adoptively transferred the cells into naïve *Myd88* ^-/-^, IL-1R^-/-^, TLR2, 3, 4, 7, 9^-/-^, TLR3, 7, 9^-/-^ and wild type mice. After four weeks of rest, we enumerated tetramer-positive, gp66-specific T cells in the spleen. The number of transferred tetramer-positive CD4^+^ T cells was significantly reduced in *Myd88* ^-/-^, IL-1R^-/-^, TLR2, 3, 4, 7, 9^-/-^ and TLR3, 7, 9^-/-^ mice vs. wild type controls (Fig 7A and 7B). These results are similar to those in the fungal vaccine model and suggest a global role of extrinsic MyD88 and upstream TLR379 in promoting the survival of CD4^+^ T cells upon activation.

## Discussion

T cell memory is the cornerstone of vaccination and vaccine-induced immunity to anti-microbial infection including fungi. Thus, understanding the mechanisms that govern long-term survival of activated T cells is germane for the rational design of vaccines. We previously reported that MyD88 is required for acquisition of anti-fungal vaccine immunity and development of protective Th17 cells [14]. Herein, we dissected where, when and how MyD88 regulates the generation of vaccine-induced anti-fungal Th17 cells.

Here, we describe a novel mechanism by which MyD88 extrinsically regulates the generation of vaccine induced T cell immunity and resistance. Our current understanding of how MyD88 affects adaptive T cell responses to fungi and other microbes is as follows. Extrinsic MyD88 signaling within APCs is thought to regulate the production of priming cytokines for Th1 and Th2 cells [20,21,22,23], whereas MyD88 within T cells can intrinsically regulate their survival and differentiation [29]. Our results demonstrate that MyD88 and TLR 3, 7, and 9 signaling extrinsically regulate the survival of activated T cells during the contraction phase, but not the expansion or priming phase. Moreover, extrinsic MyD88 was unexpectedly dispensable during the expansion and differentiation of vaccine-induced anti-fungal CD4^+^ T cells in our model and myeloid CD11c^+^ cells were dispensable for the survival of activated T cells during the contraction phase implying that the stroma might be the source of MyD88 dependent survival.

T cell intrinsic expression of MyD88 was largely dispensable for the generation of vaccine-induced Th17 and Th1 cells as assessed by the development of adoptively transferred *Myd88* ^-/-^ and *Myd88* ^+/+^ 1807 T cells in wild type recipients, and of endogenous CD4^+^ T cells in *Myd88*∆T mice. These results contrast with the intrinsic requirement of MyD88 during the priming of *B*. *dermatitidis* vaccine-induced Tc17 (CD8^+^ T cells producing IL-17) cells [30]. In the absence of CD4^+^ T cells, intrinsic MyD88 signals were indispensable for Tc17 cell responses, whereas Tc1 cells were less affected in the absence of these signals. MyD88 also has a T cell-intrinsic role for responses during LCMV, Vaccinia and *Toxoplasma* infections [29,43,44]. In contrast to the above findings, we found here that a loss of T cell-extrinsic MyD88 signals, rather than intrinsic signals, was largely responsible for the lack of protective CD4^+^ Th17 and Th1 cells during pulmonary recall and resistance to fungal challenge.

By using adoptive transfer of wild type 1807 T cells into *Myd88* ^-/-^ and *Myd88* ^+/+^ mice, we pinpointed the exact stage at which MyD88 regulates the development of Ag-specific T cells following vaccination. Extrinsic MyD88 regulated the contraction and was required for the survival of activated T cells during that phase, whereas it was dispensable for expansion, proliferation and differentiation of vaccine induced Th17 and Th1 cells. This conclusion is supported by the fact that absence of MyD88 in adoptive transfer recipients during the initial expansion and programming phase enabled the priming and differentiation of 1807 T cells, whereas absence during the maintenance phase led to accelerated contraction of 1807 cells. These results imply that extrinsic MyD88 provided a survival signal during non-inflammatory, resting conditions after adoptive transfer of effector cells into naïve recipient mice. The fact, that MyD88 regulates the survival of activated T cells under homeostatic conditions, in the absence of inflammation sets our findings apart from previously published studies in which LPS induced inflammation mediated by TLR4 signaling also promoted T cell survival [45]. LPS-TLR4-MyD88 dependent pro-memory signals were also reported for CD8^+^ T cells [46] that have different requirements than CD4^+^ T cells for T cell activation, proliferation, and differentiation and do not require MyD88 for survival in our vaccine system [30].

The mechanisms that guide transition from effector cells to memory cells are not well characterized. During the priming phase, T cell memory frequencies can be determined by the clonal burst size [47] and inflammatory signals [48,49,50] or duration of antigen availability [51,52]. During the contraction and maintenance phases, members of the IL-2 family and type I interferons IFN-α/β prevent the death of activated T cells [37,38,39,53,54]. IL-2 and its relatives induce Bcl-2 synthesis and proliferation in responsive T cells [37,38,53,54]. IFN-α/β do not increase Bcl-2 levels in T cells to stimulate T cell division [39]. IFN-α/β act directly on the T cells and not indirectly through the induction of other molecules like IL-15 and Bcl-X. We did not find evidence of differential expression of the IL-2 family members IL-7 and IL-15 and type I IFN-α/β IFNAR1, IFNAR2, IRF8 and IRF7 by naïve splenocytes and lymph node cells from *Myd88* ^-/-^, TLR3, 7, 9^-/-^ and wild type mice and the anti-apoptotic factors Bcl-2 and Bcl-xL by wild type 1807 T cells transferred into *Myd88* ^-/-^ and *Myd88* ^+/+^ recipients over the course of the priming and contraction phase. Since enhanced T cell contraction does not occur when MyD88 is absent in CD11c^+^ cells, our data are compatible with the idea that non-myeloid stromal cells could play a role in MyD88 mediated survival of activated T cells.

Since activated T cells underwent enhanced contraction in the absence of extrinsic MyD88, we investigated whether T cells showed enhanced apoptosis. We found that 1807 T cells from vaccinated *Myd88* ^-/-^ vs. wild type recipients showed increased expression of active caspase 3, but not caspase 8. Since caspase 3 is an effector caspase that funnels signals from the extrinsic and intrinsic apoptosis pathways [55], we deleted the signature molecules Fas (CD95; extrinsic) or Bim (intrinsic) from these pathways in adoptively transferred 1807 cells by crossing Bim^-/-^ and Fas^-/-^ mice with 1807 mice. Knocking out Fas or Bim in 1807 cells did not repair the exaggerated contraction in vaccinated *Myd88* ^-/-^ mice, indicating that MyD88 either does not mediate enhanced contraction through these two apoptotic pathways, or that the pathways are redundant.

By using adoptive transfer of 1807 cells and enumerating endogenous calnexin-specific T cells in knockout recipients, we identified the receptors upstream of extrinsic MyD88 mediated T cell survival. In the absence of TLRs 3, 7 and 9, activated T cells underwent enhanced contraction similar to that in *Myd88* ^-/-^ mice. The engagement of TLR3, 7 and 9 distinguishes our findings from a previous report in which LPS induced TLR4 signaling promoted T cell survival under inflammatory conditions [45]. Impaired T cell survival also correlated with the loss of vaccine-induced resistance in the corresponding knockout mice, indicating that our mechanistic analysis is functionally relevant. While others have reported that TLR signaling on T cells or myeloid cells can promote the survival of the respective cells, we are unaware of stromal cell-derived, TLR-mediated survival factors that rescue activated T cells from death. For example, T cell intrinsic MyD88 signaling has been shown to promote T cell survival in LCMV and *T*. *gondii* infection models [28,29,56]. TLRs on T cells can promote survival of activated T cells and thereby directly modulate adaptive immune responses without APC. Treatment of purified, activated CD4^+^ T cells with the dsRNA synthetic analog poly(I:C) and CpG DNA, respective ligands for TLR3 and TLR9, directly enhanced T cell survival without augmenting proliferation. Enhanced survival was associated with Bcl-xL up-regulation [57]. Porin of *Shigella dysenteriae* type 1 upregulates TLR2 to activate mitogen-activated protein kinase (MAPK) and NF-κB to induce T cell expansion by promoting both proliferation and survival of CD4^+^ T cells. The proliferation, survival and effector function of CD4^+^ T cells through TLR2 co-stimulation show the ability of porin to directly call T cells into action [58].

Collectively, we show that MyD88 signals extrinsically regulate anti-fungal vaccine immune responses by preventing apoptosis of activated CD4^+^ T cells, and that these signals likely originate with upstream TLR 3, 7 and 9, which likewise preserve the memory pool of T cells by stemming exaggerated contraction. Our findings may guide the rational design of vaccines against fungal and other microbial infections.

## Materials and Methods

### Mouse strains

Inbred wild type C57BL/6, IL-1R1^-/-^ B6.129S7-*Il1r1* ^*tm1Imx*^/J mice (stock # 003245) [59] and congenic B6. PL-Thy1^a^/Cy (stock #00406) mice carrying the Thy 1.1 allele were obtained from Jackson Laboratories, Bar Harbor, ME. *Blastomyces*-specific TCR Tg 1807 mice were generated in our lab and were backcrossed to congenic Thy1.1^+^ mice as described elsewhere [60]. *Myd88* ^-/-^ x 1807 mice that carry the congenic marker Thy1.1 were generated by crossing Thy 1.1^+^ 1807 mice with *Myd88* ^-/-^ mice twice. TLR24^-/-^, TLR379^-/-^ and TLR23479^-/-^ [61] mice were a generous gift from Dr. Carsten Kirschning from the University of Duisburg-Essen in Germany and were bred at our facility. *Myd88* ^-/-^ [62] and *Myd88*∆T mice in which MyD88 expression is selectively deleted in all αβT cells [35] were a generous gift from Drs. Doug Golenbock and Laurence Turka. All mice were 7–8 weeks at the time of experiments. All mice above were housed and cared for according to guidelines of the University of Wisconsin Animal Care Committee, who approved all aspects of this work. CD11cCre^+^ *Myd88* ^fl/fl^ [63] and CD11cCre^-^ *Myd88* ^fl/fl^ were housed in specific-pathogen free conditions at the University of California at San Francisco (UCSF).

### Enrichment, staining and analysis of rare epitope-specific T cells

To enrich epitope-specific T cells in mice we generated an anti-Cnx-specific MHC class II tetramer [42] and used a magnetic bead-based procedure that results in about a 100-fold increase in the frequency of the target population [64,65,66]. Enriched cells were stained with a cocktail of fluorochrome-labeled antibodies specific for B220, CD11b, CD11c, F4/80, CD3, CD8, CD4 and CD44. The entire stained sample was collected on an LSRII flow cytometer and live cells analyzed by FlowJo software (Treestar) following the gating strategy described [66]. The total number of tetramer positive cells from a mouse was calculated from the percent of tetramer-positive events multiplied by the total number of cells in the enriched fraction as described [66] and in the enriched plus unbound fraction when larger numbers of tetramer positive cells are present.

### Vaccination and infection

Mice were vaccinated as described [10] twice, two weeks apart, subcutaneously (s.c.) with 10^6^ to 10^7^ live or heat killed BAD1 null *B*. *dermatitidis* yeast strain #55 [67], 10^7^ live *H*. *capsulatum* strain G217B or 5 x 10^4^ live attenuated vaccine strain (∆T) that lacks the chitinases 2,3 and D-arabinotol-2-dehydrogenase as described previously [8]. Mice were infected intratracheally (i.t.) with 2 x 10^3^ or 2 x 10^4^ wild-type yeast of *B*. *dermatitidis* strain 26199, 2 x 10^5^ *H*. *capsulatum* G217B, 2 x 10^5^ FKS or 100 spores of the virulent *C*. *posadasii* isolate C735 [10,14]. To assess the infiltration of primed CD4 T cells into the lungs, challenged mice were analyzed at day 4 post-infection. To analyze the extent of lung infection, homogenized lungs were plated and yeast colony forming units (CFU) enumerated on BHI agar (Difco, Detroit, MI), sheep-blood containing Mycosel plates, or GYE plates containing 50 μg/ml of chloramphenicol [68].

### Adoptive transfer of 1807 cells and experimental challenge

To assess the T helper cytokine phenotype of Calnexin-specific CD4^+^ T cells after vaccination we purified and transferred 10^6^ CD4^+^ T cells from naïve 1807 Tg cells into C57BL/6 wild-type mice before vaccination. On the same day, recipients were vaccinated, and challenged four weeks post-vaccination.

### Generation and adoptive transfer of LCMV specific CD4^+^ effector cells

Congenic Thy1.1^+^ wild type mice were infected with 10^5^ pfu of LCMV Armstrong. 8 days later, CD4^+^ T cells were purified from the spleen and adoptively transferred into naïve *Myd88* ^-/-^, IL-1R^-/-^, TLR23479^-/-^, TLR379^-/-^ and wild type Thy1.2^+^ mice. After 4 weeks of rest the number of GP66-specific CD4^+^ T cells were assessed by tetramer that were generously provided by the NIH tetramer core facility.

### Isolation of effector cells at the site of vaccination

Mice were vaccinated once s.c. with 10^7^ heat-killed strain #55 yeast at a single dorsal site. On day 10 post-vaccination inflamed s.c. tissue was excised from the site of vaccination, placed in ice-cold collagenase buffer and minced into fine pieces. To digest the tissue, 5 ml of dissociation buffer (0.025 mg/ml Liberase (Roche Diagnostics) and 50 μg/ml DNAse I (Sigma-Aldrich) in collagenase buffer) was added and samples were incubated at 37°C and 5% CO_2_ for 30 minutes. To further release single cells, the tissue was mashed with the back of a 10-ml syringe plunger through a 70-μm cell strainer with an additional 5 ml of dissociation buffer and incubated for another 30 minutes. The dissolved tissue was washed with ice-cold PBS containing 5 mM EDTA and 1% BSA and strained again. Filtered cells were spun at 1500 rpm, the supernatant was carefully aspirated and cells were resuspended in complete media for stimulation.

### Intracellular staining

Lung cells were harvested at day 4 post-infection. Cells (0.5 x 10^6^ cells/ml) were stimulated for 4 to 6 hours with anti-CD3 (clone 145-2C11; 0.1μg/ml) and anti-CD28 (clone 37.51; 1μg/ml) in the presence of Golgi-Stop (BD Biosciences). Stimulation with fungal ligands yielded comparable cytokine production by transgenic T-cells compared to CD3/CD28 stimulation as shown previously [42,60]. After cells were washed and stained for surface CD4 and CD8 using anti-CD4 PerCP, anti-CD8 PeCy7, and anti-CD44-FITC mAbs (Pharmingen), they were fixed and permeabilized in Cytofix/Cytoperm at 4°C overnight. Permeabilized cells were stained with anti-IL-17A PE and anti-IFN-γ Alexa 700 (clone XMG1.2) conjugated mAbs (Pharmingen) in FACS buffer for 30 min at 4°C, washed, and analyzed by FACS. Cells were gated on CD4 and cytokine expression in each gate analyzed. The number of cytokine positive CD4^+^ T cells per organ was calculated by multiplying the percent of cytokine-producing cells by the number of CD4^+^ cells. Intracellular Caspase 3 Alexa647 (9602S, Cell Signaling), Caspase 8 PE (12602S Cell Signaling), Bcl2 Alexa 647 (633510 Biolegend) and Bcl-xL PE (13835 Cell Signaling) were stained from ex vivo derived lymph node cells and splenocytes.

### Cytokine protein measurements of *in vivo* primed T cells

Cell-culture supernatants were generated in 24-well plates in 1 ml containing 5 x 10^6^ splenocytes and lymph node cells and 5 μg/ml of *Blastomyces* CW/M antigen [10]. Supernatant was collected after 72 hours of co-culture. IFN-γ and IL-17A were measured by ELISA as above.

### Statistical analysis

Differences in the number and percentage of activated, proliferating or cytokine-producing T cells were analyzed using the Wilcoxon rank test for nonparametric data or the *t*-test (using GraphPad Prism) when data were normally distributed [69]. A *P* value < 0.05 is considered statistically significant.

### Ethics statement

The studies performed were governed by protocols M00969 and AN101733 as approved by the IACUC committees of the University of Wisconsin-Madison Medical School and University of California at San Francisco (UCSF), respectively. Animal studies were compliant with all applicable provisions established by the Animal Welfare Act and the Public Health Services (PHS) Policy on the Humane Care and Use of Laboratory Animals.

## Supporting Information

S1 Fig Adoptive transfer scheme, IL-7 and IL-15 transcripts and the role of CD11c+ in Myd88 regulated T cell contraction. **(A)** Purified CD4^+^ T cells from naïve Bim^-/-^, Fas^-/-^ and wild type 1807 mice were transferred into *Myd88* ^-/-^ and wild type recipient mice prior to vaccination. **(B)** IL-7 and IL-15 transcripts harvested from the skin draining lymph nodes and spleen of naïve *Myd88* ^-/-^, *TLR3*, *7*, *9* ^-/-^ and *TLR2*, *3*, *4*, *7*, *9* ^-/-^ mice vs. wild type controls. **(C)** Relative expression of IFN-α, IFN-β, IFNAR1, IFNAR2, IRF7 and IRF8 of Myd88^-/-^ mice vs. wild type mice. **(D)** Relative IRF7 expression in splenocytes from Myd88^-/-^, TLR2,3,4,7,9^-/-^, TLR2,4^-/-^ and TLR3,7,9^-/-^ vs. wild type mice. **(E)** IRF7 expression in negatively enriched pDC from Myd88^-/-^, TLR2,4^-/-^ and TLR3,7,9^-/-^ vs. wild type mice. * *P* < 0.05 vs. corresponding wild type recipient mice. **(F)** CD11c^+^ cells do not mediate MyD88 regulated T cell contraction. Effector 1807 T cells were primed in wild type recipient mice vaccinated with heat inactivated *B*. *dermatitidis* vaccine yeast as outlined in Fig 4A. CD4-purified effector T cells were adoptively transferred into naïve CD11cCre^+^-*Myd88* ^*fl/fl*^ and CD11cCre^—^ *Myd88* ^*fl/fl*^ mice and rested for four weeks. The numbers of Thy1.1^+^ 1807 T cells from the skin draining lymph nodes and the spleen were enumerated by FACS. Data are the mean ± SEM (n = 5–7 mice/group). Data are the average of two independent experiments.
(TIF)

S2 Fig TLR3,7,9 is required for T cell survival and IL-1R for Th17 cell differentiation. Purified CD4^+^ T cells from naïve 1807 mice were transferred into *Myd88* ^-/-^, IL-1R^-/-^, TLR2, 3, 4, 7, 9^-/-^, TLR3, 7, 9^-/-^ and TLR2, 4^-/-^ and wild type mice prior to vaccination. After vaccination and challenge (at day 4 post-infection), activated and cytokine-producing T cells in the lung were enumerated by FACS. **(A)** Data are expressed as the mean ± SD of 4–6 mice/group. Data are from single experiments representative of three independent experiments. * *P* < 0.05 vs. wild type control mice. **(B)** The dot plots show the sum of concatenated events from 4–6 mice/group and the values indicate the mean number of 1807 CD4^+^ T cells. Data are expressed as the mean ± SD of 4–6 mice/group from a single experiment representative of three independent experiments. * *P* < 0.05 vs. wild type control mice. **(C)** The frequencies of cytokine producing 1807 T cells in IL-1R^-/-^ and wild type mice that were vaccinated or not from Fig 6B. * *P* < 0.05 vs. wild type control mice. **(D)** Resistance to *C*. *posadasii* infection. TLR2, 3, 4, 7, 9^-/-^, TLR3, 7, 9^-/-^ and wild type mice were vaccinated with 5 x 10^4^ live attenuated (∆T) strain or not. Seven weeks later, mice were challenged with 10^2^ spores of *C*. *posadasii* strain C735 and the number of CFU determined at two weeks post-infection.
(TIF)
