# Supplementary figures and images for: MyD88 Shapes Vaccine Immunity by Extrinsically Regulating Survival of CD4+ T Cells during the Contraction Phase
Source: PLoS Pathog. 2016 Aug 19;12(8):e1005787. doi: 10.1371/journal.ppat.1005787 (PMC4991787; doi:10.1371/journal.ppat.1005787)

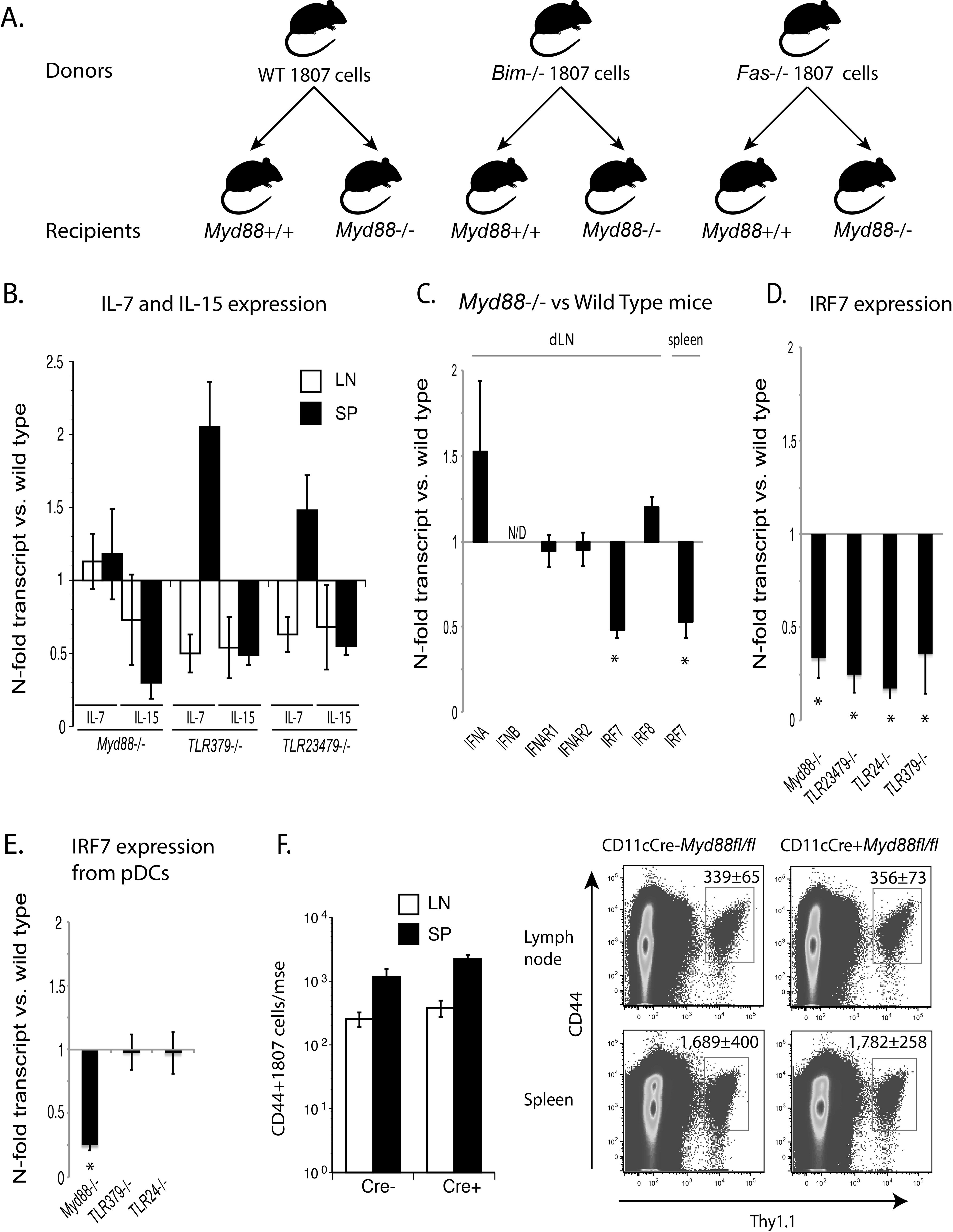

Supplement: S1 Fig — (A) Purified CD4+ T cells from naïve Bim-/-, Fas-/- and wild type 1807 mice were transferred into Myd88 -/- and wild type recipient mice prior to vaccination. (B) IL-7 and IL-15 transcripts harvested from the skin draining lymph nodes and spleen of naïve Myd88 -/-, TLR3, 7, 9 -/- and TLR2, 3, 4, 7, 9 -/- mice vs. wild type controls. (C) Relative expression of IFN-α, IFN-β, IFNAR1, IFNAR2, IRF7 and IRF8 of Myd88-/- mice vs. wild type mice. (D) Relative IRF7 expression in splenocytes from Myd88-/-, TLR2,3,4,7,9-/-, TLR2,4-/- and TLR3,7,9-/- vs. wild type mice. (E) IRF7 expression in negatively enriched pDC from Myd88-/-, TLR2,4-/- and TLR3,7,9-/- vs. wild type mice. * P < 0.05 vs. corresponding wild type recipient mice. (F) CD11c+ cells do not mediate MyD88 regulated T cell contraction. Effector 1807 T cells were primed in wild type recipient mice vaccinated with heat inactivated B. dermatitidis vaccine yeast as outlined in Fig 4A. CD4-purified effector T cells were adoptively transferred into naïve CD11cCre+-Myd88 fl/fl and CD11cCre— Myd88 fl/fl mice and rested for four weeks. The numbers of Thy1.1+ 1807 T cells from the skin draining lymph nodes and the spleen were enumerated by FACS. Data are the mean ± SEM (n = 5–7 mice/group). Data are the average of two independent experiments. (TIF) [file ppat.1005787.s001.tif]

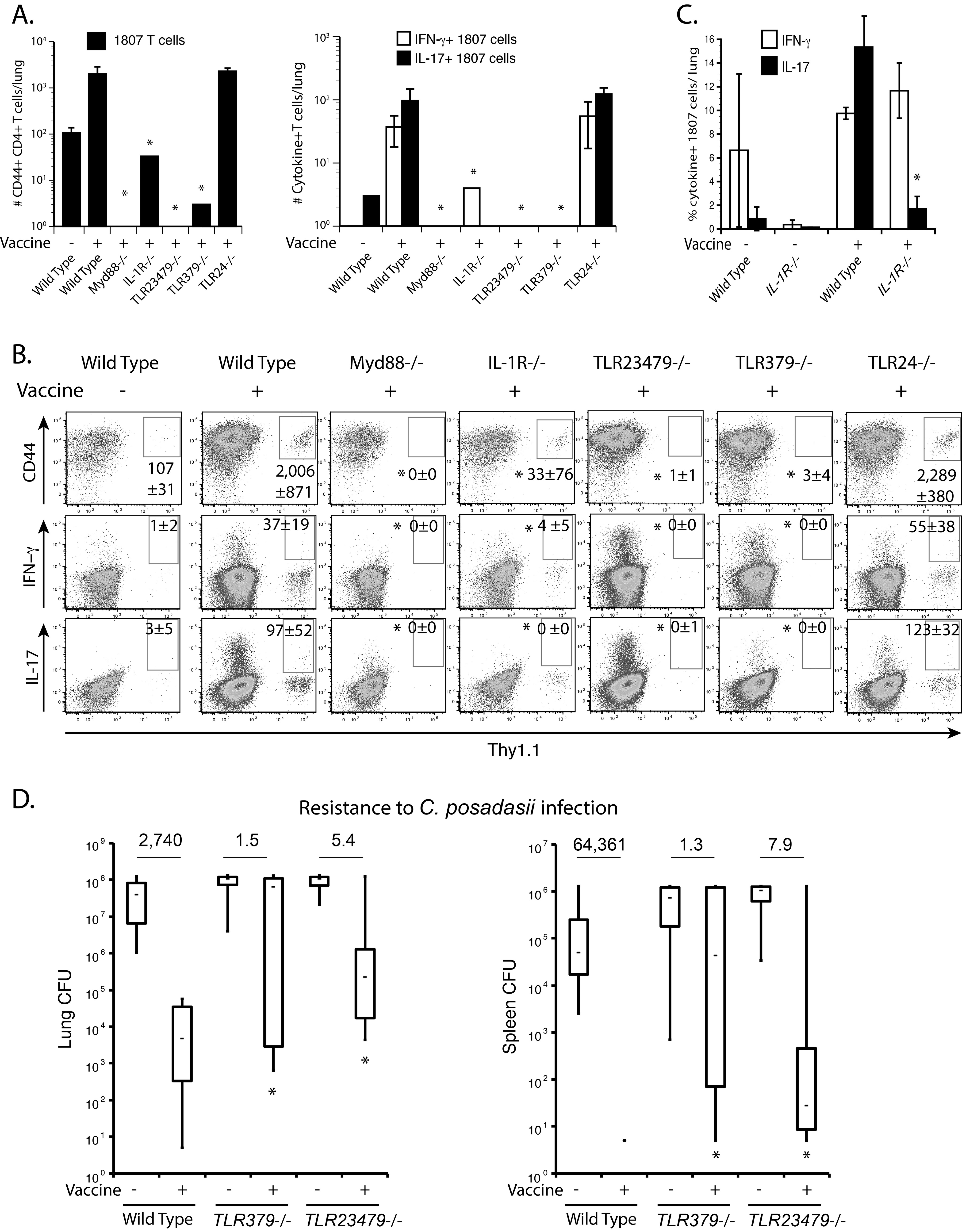

Supplement: S2 Fig — Purified CD4+ T cells from naïve 1807 mice were transferred into Myd88 -/-, IL-1R-/-, TLR2, 3, 4, 7, 9-/-, TLR3, 7, 9-/- and TLR2, 4-/- and wild type mice prior to vaccination. After vaccination and challenge (at day 4 post-infection), activated and cytokine-producing T cells in the lung were enumerated by FACS. (A) Data are expressed as the mean ± SD of 4–6 mice/group. Data are from single experiments representative of three independent experiments. * P < 0.05 vs. wild type control mice. (B) The dot plots show the sum of concatenated events from 4–6 mice/group and the values indicate the mean number of 1807 CD4+ T cells. Data are expressed as the mean ± SD of 4–6 mice/group from a single experiment representative of three independent experiments. * P < 0.05 vs. wild type control mice. (C) The frequencies of cytokine producing 1807 T cells in IL-1R-/- and wild type mice that were vaccinated or not from Fig 6B. * P < 0.05 vs. wild type control mice. (D) Resistance to C. posadasii infection. TLR2, 3, 4, 7, 9-/-, TLR3, 7, 9-/- and wild type mice were vaccinated with 5 x 10^4^ live attenuated (∆T) strain or not. Seven weeks later, mice were challenged with 102 spores of C. posadasii strain C735 and the number of CFU determined at two weeks post-infection. (TIF) [file ppat.1005787.s002.tif]
